# Climate change and crop resilience: harnessing metabolomics for predicting stress tolerance

**DOI:** 10.1111/nph.71219

**Published:** 2026-05-01

**Authors:** Agyeya Pratap, Arash Fazeli, Ali Bandehagh, Nicolas L. Taylor

**Affiliations:** ^1^ UWA Node of Australian Plant Phenomics Network, School of Molecular Sciences, ARC Training Centre in Predictive Breeding for Agricultural Futures, ARC Centre of Excellence in Plant Energy Biology, and The UWA Institute of Agriculture, The University of Western Australia Boorloo WA 6009 Australia; ^2^ Agronomy and Plant Breeding Department, Faculty of Agriculture Ilam University Ilam Ilam Province 69315‐516 Iran

**Keywords:** abiotic stress tolerance, biomarkers, climate resilience, crop improvement, genomic prediction, metabolomics, multi‐omics integration, yield

## Abstract

Global warming is driving climate change to levels not experienced since the advent of agriculture, primarily due to anthropogenic factors and the accumulation of CO_2_. Rising temperatures, frequent droughts, and elevated CO_2_ levels are reducing crop productivity in key agricultural regions. Developing climate‐resilient crop varieties is essential. Metabolomics provides a powerful tool for quantifying plant responses to abiotic stressors and identifying predictive biomarkers for stress tolerance. While metabolite‐based diagnostics are well‐established in clinical research, their integration into crop breeding remains limited. This Tansley review highlights recent advances in metabolomics for predicting yield stability and quality under stress, emphasising the role of metabolic biomarkers in resolving complex genotype × environment interactions. We discuss the utility of metabolite quantitative trait loci, metabolome‐wide association studies, and machine learning‐driven metabolic marker‐assisted genomic prediction in enhancing trait prediction. These approaches complement genomic selection, improving accuracy and resilience forecasting. We also address methodological challenges in translating metabolomics into breeding pipelines, including standardisation and data integration. By combining metabolomics with genomics, modelling, and high‐throughput phenotyping, researchers can accelerate the development of stress‐resilient crops. This Tansley review presents a framework for leveraging metabolomics in predictive breeding, offering a transformative pathway toward sustainable agriculture in a changing climate.

## Graphical abstract

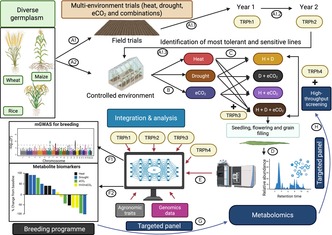

**Table:** 

Contents		
	Summary	975
I.	Introduction	976
II.	Climate change drivers and their direct effects on crops	976
III.	Impacts on the plant metabolome	978
IV.	Quantitative biomarker panels for stress prediction	987
V.	Advanced prediction models for stress tolerance	989
VI.	Conclusion and future perspectives	991
	Acknowledgements	991
	References	992

## I. Introduction

### 1. The plant metabolome

Metabolomics has emerged as a powerful tool for elucidating the biochemical responses of plants to environmental stresses. By capturing the comprehensive dynamics of metabolites, it enables the identification of stress‐responsive biomarkers and provides mechanistic insights into how plants adapt to abiotic stresses. When integrated with other omics technologies, metabolomics offers a system‐level perspective, linking metabolic changes in gene expression and phenotypic outcomes (Katam *et al*., 2022). Plants exhibit a high degree of metabolic plasticity due to their exposure to dynamic spatial, temporal, and environmental conditions (Salam *et al*., 2023). This complexity renders plants ideal models for investigating metabolic networks and pathways. By utilising both reverse and forward genetics, including genome sequencing and population genetics, researchers can dissect the molecular basis of stress response and uncover key metabolic adaptation mechanisms (Fang *et al*., 2019; Zandalinas *et al*., 2022).

### 2. Scope and objectives

This Tansely review focuses on the application of metabolomics for developing predictive biomarkers to infer stress tolerance of crops under stress. Metabolomics facilitates the identification of stress‐responsive metabolite biomarkers, which act as indicators of physiological and biochemical responses in plants. These biomarkers are increasingly being employed in breeding programmes to improve resilience. Advanced approaches, such as metQTL mapping, metabolome‐wide association studies (mGWAS), and machine learning (ML)‐driven metabolic marker‐assisted genomic prediction (MM_GP), enhance the precision of stress tolerance assessments (Lee *et al*., 2025). This Tansley review also addresses the technical and methodological challenges in translating metabolomics data into actionable breeding pipelines, including the need for standardised methodologies and predictive modelling. By highlighting key innovations in biomarker discovery and translational metabolomics, we present a framework for leveraging these tools in the development of climate‐resilient crops.

### 3. Challenges in linking molecular and physiological data

A key objective in modern agriculture is to ensure stable crop production in the face of increasing climate variability. Two primary strategies are employed to achieve this: introducing stress tolerance traits into elite cultivars and improving yields in underutilised or wild crop species (Tardieu, 2012). Although numerous stress‐responsive genes have been identified through functional genomics, translating these into improved field performance remains a bottleneck (Palmgren & Shabala, 2024). A critical challenge is the disconnect between molecular‐level responses, such as changes in metabolite concentrations, and whole‐plant physiological traits. While physiological parameters (e.g. photosynthetic efficiency, water use) are measured over long timeframes and large spatial scales, metabolite changes are often tissue‐specific and occur rapidly (Obata & Fernie, 2012). This temporal and spatial mismatch complicates efforts to link molecular profiles with phenotypic outcomes (Weckwerth, 2011). For example, common stress‐associated metabolites, such as proline and sugars, often accumulate during stress; however, their presence does not consistently correlate with yield improvements under field conditions (Obata & Fernie, 2012). Genotype × environment interactions and variability in environmental conditions further obscure these relationships, limiting the reproducibility and reliability of metabolomic markers across studies (Libbrecht & Noble, 2015). Inconsistencies in sampling protocols, instrumentation, and data processing in metabolomics often hinder cross‐comparison, whereas physiological measurements, such as stomatal conductance or Chl fluorescence, are typically better standardised. Although ML offers promise in associating large omics datasets with phenotypic traits, model overfitting remains a concern, limiting generalisability across different environments or genotypes (Tardieu *et al*., 2017). Emerging approaches that integrate omics data with time‐resolved physiological measurements could help bridge this gap, offering a more holistic view of plant responses to stress (Raza *et al*., 2025). While metQTL mapping, mGWAS and MM_GP provide essential tools for understanding genotype × environment interactions in crops, these approaches require extensive datasets, standardised methods, and advanced computational analysis.

### 4. Role of technological advancements in omics studies

Climate change stress tolerance prediction in crops is being revolutionised by developments in high‐throughput phenomics (HTP), metabolomics, and multi‐omics integration. With the precision and accuracy of metabolic profiling improvements in nuclear magnetic resonance (NMR) spectroscopy and mass spectrometry (MS), researchers can more confidently identify stress‐responsive biomarkers (Bhinderwala *et al*., 2018). The integration of metabolomics with molecular breeding techniques, such as metQTL and mGWAS, facilitates the identification of gene expression linked to the abundance of specific metabolites. The integration of metabolomics data into GP models, through MM_GP, incorporates essential abundance data to enhance the accuracy of GPs in crop breeding (Hu *et al*., 2021; Wu *et al*., 2022; Xu *et al*., 2025). Advancements in technology are enhancing plant phenomics, a field that combines imaging, molecular techniques, and environmental monitoring (Xu *et al*., 2025). Integrating metabolomics and HTP using artificial intelligence (AI) presents an opportunity for researchers to better understand plant biology, stress responses, and improve breeding for higher yield and resilience. These innovations are making it easier to translate metabolomics data into actionable breeding strategies, accelerating the development of stress‐resilient crop varieties.

## II. Climate change drivers and their direct effects on crops

Our planet is undergoing a significant transformation due to global warming‐driven climate change, a consequence of human activities. These alterations are impacting biodiversity, food security, and public health (Pörtner *et al*., 2021). With the global population expected to grow to 9.7 billion by 2050, the demand for food has escalated, with projections indicating a 30–62% increase over the total food needs recorded in 2010 (van Dijk *et al*., 2021). However, food production is facing growing pressure due to the combined effects of climate change and the rapid urban expansion of agricultural land (Lenaerts *et al*., 2019). In recent years, the accelerated pace of climate change has raised concerns among scientists, farmers, and leaders about the threats to food supply, a situation further worsened by recent wars, antimigration policies, and trade disruptions (Rhee *et al*., 2025). Understanding how climate change impacts crop production is crucial for forecasting and mitigating its impact on political, economic, health, and food security.

### 1. Drought stress

Drought stress, driven by decreases in seasonal rainfall in previously productive agricultural regions, limits plant growth through decreases in photosynthesis and respiration (Manna *et al*., 2021; Heilemann *et al*., 2024). For example, in wheat, water deficit causes a significant reduction in growth and yield by impairing stomatal conductance and water use efficiency (Zulfiqar *et al*., 2024), while in soybean genotypes, water deficit causes decreased photosynthetic rates, water use efficiency, and overall yield losses (Hossain *et al*., 2024). Plant tissues respond differently to drought stress during both vegetative and reproductive growth stages, and plants alter their morphology and employ escape, avoidance, and resistance strategies (Vadez *et al*., 2024; Raza *et al*., 2025). The expression of transcription factors and genes can influence drought stress tolerance. For example, the upregulation of a particular cytokinin (CK) oxidase/dehydrogenase (*MiCKX4*) has been associated with the elongation of the primary root and an increase in the number of lateral roots, which has been shown to mitigate drought stress (Singhal *et al*., 2024). *PeHDZ72*, a member of the HD‐Zip transcription factor (TF) family, is highly expressed in *Phyllostachys edulis* (Barnabás *et al*., 2008), inducing *PeSWEET_23178* and *PeTIP4‐*3, which are also upregulated in response to drought (Zhu *et al*., 2025). In maize, the *ZmWRKY71* gene shows increased expression in roots under drought conditions (Ma *et al*., 2025). As drought poses a formidable challenge to the sustainability of global food production, there is an urgent need to develop drought‐tolerant crop varieties.

### 2. Heat stress

Global warming has led to an increase in the frequency of heatwaves, which interfere with crop growth, reducing yields, and crop productivity. For example, in wheat, terminal heat stress severely impacts wheat productivity by decreasing grain yield and number (Ullah *et al*., 2022), while pre‐anthesis heat stress yields mixed results for grain weight while consistently decreasing grain yield, biomass, harvest index, and grain number (A. S. Talukder *et al*., 2014; S. K. Talukder *et al*., 2014; Balla *et al*., 2019; Choudhary *et al*., 2022; Xu *et al*., 2022). Postanthesis heat stress notably reduces grain weight, biomass, reproductive tillers, grain number, harvest index, and grain yield, while increasing grain protein content (Spiertz *et al*., 2006; A. S. Talukder *et al*., 2014; S. K. Talukder *et al*., 2014; Bergkamp *et al*., 2018). Additionally, heat stress during grain filling significantly reduces grain quality in wheat, causing further economic losses (Mahdavi *et al*., 2022). Heat stress during wheat ear emergence and grain filling reduces grain number and weight and tolerant genotypes maintain photosynthesis, pollen viability, and redox balance, with proteomic evidence linking heat tolerance to proteins involved in photosynthesis, metabolism, and redox homeostasis (Pratap *et al*., 2025). In rice, heat stress severely impacts pollen germination and viability, as well as ovary development and starch accumulation in developing grains (Zhang *et al*., 2018). When heat stress occurs during grain filling, it reduces rice grain weight (Shi *et al*., 2017), yield (Folsom *et al*., 2014), and quality by increasing chaffiness (Amjadi *et al*., 2024). In maize, heat stress severely affects vegetative growth and development (Crafts‐Brandner & Salvucci, 2002). During the flowering period, it can have even more detrimental effects on seed set (Shao *et al*., 2021) and biomass and yield (Carter *et al*., 2016). In tomatoes, heat stress reduces the number of open flowers and floral resources, resulting in lower yields (Defalque *et al*., 2025). Similarly, in cotton, significant declines in net photosynthesis and yield are observed (Chastain *et al*., 2024). Overall, heat stress due to global warming poses a significant threat to global crop production and food security by decreasing photosynthesis, pollen viability, floret fertility, yield, and quality.

### 3. Elevated CO_2_

Elevated CO_2_ levels can also negatively impact crop productivity by influencing photosynthesis, water use efficiency, and nutrient uptake. While elevated CO_2_ levels can increase biomass, they often lead to nutrient imbalances and a reduction in grain quality. For example, biochar application under elevated CO_2_ conditions increases soil temperatures and biomass in durum wheat but does not alleviate nitrogen reduction in grains, resulting in lower grain quality (Brilli *et al*., 2025). In soybean, elevated CO_2_ improves photosynthetic efficiency but requires careful nutrient management to avoid negative impacts on yield (Pequeno *et al*., 2021). Climate change projections suggest that elevated CO_2_ levels could increase maize yield; however, the benefits may be offset by increased temperatures and decreased rainfall under high‐emission scenarios, ultimately reducing productivity (Chen *et al*., 2024). Elevated CO_2_, a key driver of climate change, has complex effects on crop growth and productivity. While increased CO_2_ can enhance photosynthesis, particularly in C_3_ crops such as rice, wheat, and soybeans (Streck, 2005), and improve water use efficiency, these benefits depend on interactions with other environmental factors. For instance, co‐elevation of CO_2_ and temperature has been shown to improve water use efficiency in rice (Maity *et al*., 2023) and enhance growth parameters in tomato (Rangaswamy *et al*., 2021). However, high temperatures can negate the positive effects of CO_2_, reducing biomass accumulation and yield. Additionally, elevated CO_2_ can lower nutrient concentrations in plant tissues, affecting crop quality and food security (Khan *et al*., 2025), making nutrient management strategies essential.

### 4. Combined stresses and synergistic effects

Climate change is exerting significant pressure on plants, negatively impacting crop development and food security. Stress tolerance is defined as the ability of plants to persist or thrive under stressful conditions, in contrast to their growth under optimal conditions (Skirycz *et al*., 2011). The interaction of one or more climate change factors can lead to synergistic, neutralising, or dominant effects (Sun & Fernie, 2024). There are complex interactions between climate change‐induced stresses and crop productivity, highlighting the need for integrated management strategies to mitigate their adverse effects on global agriculture.

The interaction between elevated CO_2_ and other stressors, such as heat and drought stresses, significantly influences agricultural productivity. Studies indicate that heat stress can offset the yield benefits of elevated CO_2_ in wheat (Chavan *et al*., 2022) and reduce CO_2_‐induced growth improvements in bell pepper (Kumari *et al*., 2021). Additionally, elevated CO_2_ can alter crop–pest interactions, potentially increasing susceptibility to pests and diseases (Trębicki *et al*., 2017). In wheat, combination of heat and drought stress reduces growth and yield, and elevated CO_2_ consistently had lower spike biomass under combinations with heat, drought, and heat and drought stresses compared with control treatment (Milec *et al*., 2025). The interaction of these stresses affect not only just the plants but also their microbiomes creating another layer of negative effects on overall health of the ecosystem (Zandalinas & Mittler, 2022). Zandalinas *et al*. (2021) show that while individual mild stresses have little effect on plants, the simultaneous combination of several low‐level stresses causes a severe decline in plant growth and survival, and multifactorial stress triggers unique physiological and molecular responses not seen under single stresses. By understanding and addressing these abiotic stresses, we can develop more resilient agricultural systems that ensure food security in the face of a changing climate.

## III. Impacts on the plant metabolome

### 1. Primary and secondary metabolites

Studies of plant metabolic plasticity under multifactorial abiotic stresses have led to significant advances in understanding adaptation mechanisms, paving the way for the development of climate‐resilient crops. Major crops, such as wheat, rice, and maize, have been widely studied for their responses to heat, drought, elevated CO_2_, and their combinations (Table 1). In response, plants adapt their internal mechanisms and produce various compounds to manage these challenges (Zandalinas *et al*., 2022). Large‐scale metabolite catalogues, such as those generated for legumes by Bulut *et al*. (2023), highlight the extent of metabolic diversity across crop species. These environmental stressors primarily affect amino acids (proline, ɣ‐aminobutyric acid (GABA), branched chain amino acids, and arginine), sugars (trehalose, fructose, and glucose), and organic acids, which help plants tolerate stress or modulate growth (Table 1). The type and intensity of stress can influence the production of these secondary metabolites under climate change. To cope with environmental pressures such as biotic and abiotic stresses, plants produce specialised secondary metabolites that can have antioxidant properties and may accumulate osmolytes, including proteins, proline, glycine betaine, phenolic compounds, and soluble sugars. These compounds help support the survival of organisms under stress (Nakabayashi & Saito, 2015; Wahab *et al*., 2022). Overall, these metabolic adjustments and secondary metabolite accumulations form a critical defence strategy, enabling plants to maintain cellular homeostasis and survive under complex climate‐induced stresses.

**Table 1:** Metabolite abundance changes compared to ambient conditions under heat, drought, and elevated CO_2_ and their combinations.

Group	Metabolite	D	H	CO_2_	D + H	D + eCO_2_	H + eCO_2_	Plants
Sugars	Galactose							Cucumber, *Suaeda* spp.
	Maltose							Cucumber, Maize, *Triticea* spp.
	Galactinol							*Suaeda* spp., Coffee, Maize, Rice
	Starch							Maize, Rice
	Raffinose							Cucumber, Coffee, Soybeans, Rice, Maize
	Sucrose							*Salix* spp., Rice, Maize, Wheat, Canola, Cucumber, *Isatis* spp., *Suaeda* spp., Coffee
	Glucose							*Salix* spp., Coffee, Soybean, Rice, Maize, Wheat, Canola
	Mannose							Cucumber, Maize, *Triticea* spp.
	Fructose							Coffee, Soybean, Maize, Wheat, Canola
	Trehalose							Cucumber, *Isatis* spp., Coffee, Soybean, Rice, Maize, *Triticea* spp.
	Myo‐inositol							*Suaeda* spp., Maize, Coffee
	Mannitol							*Suaeda* spp., Soybean, Rice, Maize
	Sorbitol							Tomato, Soybean, Rice, Maize
Amino acids	Serine							*Suaeda* spp., Arabidopsis, Coffee, Cotton, Maize, Citrus
	Glycine							*Suaeda* spp., Arabidopsis, Coffee, Soybean, Rice, Maize
	Leucine							*Suaeda* spp., Soybean, Wheat, Rice, Maize, Arabidopsis, Sesame
	Valine							Arabidopsis, Coffee, Rice, Maize, Soybean, Sesame
	Isoleucine							*Suaeda* spp., Arabidopsis, Coffee, Soybean, Maize, Rice, Wheat, Potato
	Alanine							*Suaeda* spp., Coffee, Soybean, Rice, Maize, Canola
	Asparagine							Suaeda spp., Coffee, Soybean, Rice, Maize, Sesame
	Methionine							Coffee, Soybean
	Lysine							Coffee, Soybean, Sesame, Potato, Wheat
	Theronine							Rice, Maize, Potato
	Tryptophan							*Suaeda* spp., Coffee, Soybean, Arabidopsis, Rice, Wheat, Maize, Potato
	Phenylananine							Pepper, *Suaeda* spp., Coffee, Maize, Canola, Rice
	Tyrosine							Coffee, Soybean, Maize, Canola, Wheat
	Glutamate							Cucumber, *Suaeda* spp., Arabidopsis, Coffee, Wheat, Maize
	Glutamine							*Suaeda* spp., Wheat, Arabidopsis
	Arginine							Coffee, Sesame, Rice, Wheat, Potato
	GABA/ɣ‐amino‐butyrate							Coffee, Maize, Sesame, *Triticea* spp., Squash
	Proline							*Suaeda* spp., Coffee, Soybean, Wheat, Rice, Maize, Canola, Sesame, Citrus, Potato
	Aspartate							Coffee, Maize
	Histidine							Potato
	Cysteine							Cotton
TCA cycle metabolites	Citrate							*Suaeda* spp., *Salix* spp., Arabidopsis, Coffee, Soybean, Carrot, Rice, Maize
	2‐Oxoglutarate							Cucumber, *Salix* spp., Arabidopsis, Coffee
	Succinate							Cucumber, *Salix* spp., Coffee, Soybean, Maize, Citrus, Wheat
	Fumarate							*Salix* spp., Coffee, Sesame, Maize
	Malate							*Suaeda* spp., *Salix* spp., Arabidopsis, *Coffee*, Soybean, Rice, Maize
	Glyoxylate							Maize
	Pyruvate							Maize
Others	Putrescine							Coffee
	L‐ascorbate							Wheat
	Nicotinic acid							Coffee
	Shikimate							*Salix* spp., Maize
	(3Z)‐Phytochromobilin							Cucumber
	Lipid intermediates							Maize
Antioxidants	Phenylpropanoids							Pepper, *Salix* spp., Soybean
	Flavonoids							*Salix* spp., Soybean
Hormones	Abscisic acid							Arabidopsis, Wheat, Rice, Maize, Potato
	Auxin							Rice, Maize
	Brassinosteroids							Cucumber
	Cytokinins							Rice, Cucumber, Maize
	Jasmonic acid							Cucumber, Wheat, Potato
	Gibberlic acid							Rice, Sunflower
	Ethylene							Rice, Wheat
	Salicyclic acid							Rice, Wheat
	Strigolactones							Tomato, *Festuca arundinacea* Schreb

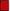
, Overaccumulation; 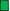, Underaccumulation; 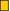, Over‐ or underaccumulation; 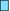, No change; 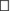, Data not available. Full references for this table are provided in Supporting Information Table S1. D, drought; H, heat; eCO_2_, elevated CO_2_; TCA, tricaboxylic acid cycle.

In the wheat cultivar Norin‐61, progressive drought induces purine and pyrimidine metabolism, 1‐aminocyclopropane‐1‐carboxylic acid, asparagine and serotonin accumulation (Table 1). Drought also impacts the metabolism of proline and glycine betaine, as well as the biosynthesis of tanshinones and phenolic acids with the *AHL* gene family regulating the production of neocryptotanshinone (Zhang *et al*., 2025). In wheat, abscisic acid (ABA) treatment increases the content of tagatose and L‐serine in *TaPYLox* overexpressing wheat, while the branched‐chain amino acids, such as valine, leucine, and isoleucine, are unaffected but are reduced in drought‐resistant varieties (Weng *et al*., 2024). The combination of these elements and pathways enhances the plant's ability to sense, respond, and adapt to drought stress.

Heat stress significantly impacts plant metabolism, triggering distinct metabolic pathways critical to stress adaptation and tolerance. In maize (Table 1), substantial metabolic plasticity is observed when exposed to elevated temperatures, revealing common responses such as accumulation of sugars, amino acids, and compatible solutes. Additionally, metabolites such as shikimate and aromatic amino acids are upregulated, indicating a reconfiguration of metabolic networks essential for adaptation (Table 1). In cucumber, comparative metabolomics distinguished thermotolerant from thermosensitive varieties, highlighting enhanced photosynthesis, Chl metabolism, and reactive oxygen species (ROS)‐scavenging pathways in heat‐tolerant plants (Table 1). Canola seedlings subjected to heat stress exhibited significant changes in their metabolic profiles, including increased levels of essential amino acids, sucrose, and a decrease in glucose and fructose, suggesting that altered carbohydrate metabolism serves as a stress‐adaptive mechanism (Table 1). Similarly, in *Brassica napus*, metabolomics uncovered temperature‐sensitive resistance mechanisms, identifying lipid metabolism, amino acid metabolism, and phenylpropanoid biosynthesis pathways as crucial for heat stress responses. Moreover, elevated temperature compromises key defence pathways, leading to reduced resistance (Amjadi *et al*., 2024). In wheat, heat stress also induces the accumulation of tryptophan and pipecolate (involved in amino acid and secondary metabolite biosynthesis), while decreases in drummondol and anthranilate (involved in ABA catabolism and tryptophan metabolism) have been observed (Thomason *et al*., 2018). There is enormous diversity in the metabolic responses of different crops and varieties to heat stress, including the accumulation of sugars, amino acids, antioxidants, and Chl, as well as lipid metabolism, providing an invaluable tool for biomarker discovery and utilisation in breeding programmes.

Elevated atmospheric CO_2_ levels play a crucial role in climate change and plant metabolism, impacting both primary and secondary metabolite profiles. The plant metabolome, which encompasses all small molecules involved in metabolic activities, responds to elevated CO_2_ by adjusting carbon allocation, nutrient absorption, and stress signalling pathways. Primary metabolites such as carbohydrates, amino acids, and organic acids play a direct role in growth and development. Plants in elevated CO_2_ increase photosynthetic activity, resulting in higher accumulation of carbohydrates such as glucose, fructose, and sucrose (Ainsworth & Long, 2005; Leakey *et al*., 2009). This carbon surplus disrupts the carbon/nitrogen balance, often leading to lower protein and amino acid concentrations due to nitrogen dilution (Taub & Wang, 2008). Amino acids such as glutamine, asparagine, and glycine tend to decrease, which can impair nitrogen metabolism and protein synthesis (Bloom *et al*., 2014). Furthermore, organic acids of the CA cycle, such as malate and citrate, show varying responses under elevated CO_2_. This may reflect metabolic adaptations to accommodate improved carbon skeleton supply for secondary metabolism. Secondary metabolites, including phenolics, flavonoids, alkaloids, and terpenoids, play crucial roles in plant defence, signalling, and adaptation to abiotic stress. Elevated CO_2_ often stimulates the biosynthesis of secondary metabolites, particularly phenolic compounds and flavonoids, due to increased substrate availability resulting from enhanced photosynthesis (Table 2). These compounds contribute to antioxidant capacity and may bolster plant resilience against oxidative stress induced by elevated CO_2_ and associated environmental changes. However, the response of secondary metabolites is highly species‐specific and can be modulated by interactions with other environmental factors such as nutrient availability, drought, and temperature (Li *et al*., 2008; Table 2). Metabolomics offers a powerful platform for unravelling these complex metabolic responses, enabling the identification of biomarkers associated with elevated CO_2_ stress tolerance.

**Table 2:** Overview of phytohormone biosynthesis, signalling, and functional roles under environmental stresses.

Hormone	Biosynthesis and storage	Core signaling components	Primary physiological roles	Crosstalk and stress‐associated notes
Abscisic acid (ABA)	Produced via carotenoid pathway. Stored as ABA‐glucose ester.	*PYR, PYL*, and *RCAR* receptors.	Stomatal behaviour, osmotic adjustment, seed dormancy.	*RCAR12/13* enhance *UGT71C5*. Interacts with JA and SA. Restores terpene abundance.
Brassinosteroids (BRs)	Derived from sterol biosynthesis via mevalonate.	*BRI1* and *BAK1* receptors. *BES1* and *BZR1* factors.	Growth, cell expansion, stress mitigation and crosstalk with ROS.	*RD26* modulates growth under drought. *OsHDAC1‐OsGSK2* stabilises *OsBZR1*. *BvBZR1* increases auxin and GA.
Auxin	Synthesised via tryptophan pathways. Regulated by polar transport.	*TIR1* and *AFB* receptors, *Aux/IAA* proteins and ARF proteins.	Shoot and root development. Antioxidant responses.	Improves grain set under heat. Maintains antioxidant capacity. Enhances drought tolerance with GA. Drives hyponasty under heat and CO_2_.
Jasmonic acid (JA)	Produced via octadecanoid pathway. Active form is JA‐Ile.	*COI1* receptor, *JAZ* repressors and *MYC* transcription factors.	Antioxidant induction, HSP expression, development, defence.	Levels rise under drought and heat. Decline under elevated CO_2_. Promotes tolerance.
Salicylic acid (SA)	Produced via phenylpropanoid and isochorismate pathways.	*NPR1, NPR3, NPR4*, and *TGA* transcription factors.	Defence gene expression. Oxidative stress.	Responses vary. Increase under heat. Decline under CO_2_. Foliar SA reduces drought damage.
Cytokinins (CKs)	Synthesised via isopentenyl transferases Modified by hydroxylation and conjugation.	Histidine kinases, phosphotransfer proteins and type‐B *RRs*.	Cell division, meristem activity, membrane stability.	Heat and drought reduce CK. Elevated CO_2_ increases CK.
Ethylene	Synthesised from methionine via SAM and ACC.	Ethylene receptors, *CTR1, EIN2, EIN3* and *ERF* proteins.	Senescence, abscission, stress signalling.	Drought and heat increase ethylene. CO_2_ suppresses ethylene. Promotes anthocyanin in drought.
Gibberellins (GA)	Produced via diterpenoid pathway from GGDP.	*GID1* receptors and *DELLA* proteins.	Stem elongation, germination, flowering. GA3 enhances drought growth.	External GA increases grain weight under heat CO_2_ offsets GA inhibitors Endogenous GA declines under stress.
Strigolactones (SLs)	Biosynthesis begins with conversion of carotenoids via *DWARF27*.	*D14* receptor and *SMXL* protein degradation.	Regulate branching, root architecture, senescence, AM symbiosis. Enhance stress resilience.	Interact with ABA, cytokinin and auxin. Regulate MAPK linked stress responses.

Full references for this table are provided in Supporting Information Table S2. ERF, Ethylene responsive factor.

Drought and heat stress induce profound changes in both primary and secondary metabolite profiles in plants, reflecting a complex adaptive response to environmental adversity. Under these stress conditions, a rapid accumulation of primary metabolites, such as soluble sugars, amino acids, and organic acids, typically occurs (Table 1). This increase serves as an immediate protective mechanism, helping to maintain osmotic balance, stabilise cellular structures, and provide energy for stress responses (Table 1). As the stress persists, the metabolic flux shifts toward the synthesis and accumulation of secondary metabolites, including phenolic compounds, flavonoids, and various antioxidants (Table 1). These secondary metabolites play crucial roles in scavenging ROS and protecting cells from oxidative damage (Table 1). The coordinated buildup of both primary and secondary metabolites not only supports immediate survival but also enhances long‐term resilience from prolonged drought and heat episodes (Chávez‐Arias *et al*., 2022; Yadav *et al*., 2021). The temporal dynamics of metabolite responses to drought and heat stress are further highlighted, with a clear distinction between the early and late phases of stress exposure. Furthermore, under combined heat and drought stress conditions, sucrose, glucose, and fructose are more likely to accumulate, while there was no consistent trend of increase or decrease in sugars, amino acids, or TCA cycle metabolites in response to the individual stressors (Zandalinas *et al*., 2022). Metabolic changes in *Arabidopsis* exposed to drought, heat, and combined stress revealed that under combined stress, plants accumulated sucrose, maltose, and glucose, whereas proline levels increased only under drought conditions (Rizhsky *et al*., 2004). Heat stress appears to reduce proline toxicity, specifically during heat stress, leading to oxidative damage rather than protection, suggesting that sucrose replaces proline as the main osmoprotectant in plants under combined stresses (Rizhsky *et al*., 2004). A recent report on Arabidopsis has synthesised current knowledge on metabolic reprogramming across major pathways, including amino acid biosynthesis, starch metabolism, glycolysis and gluconeogenesis, and flavonoid biosynthesis, under key abiotic stresses such as elevated CO_2_, salinity, drought, heat, high‐light intensity, and UV‐B radiation (Bulut *et al*., 2025a). In parallel, a comprehensive review has collated and compared metabolite responses to salinity stress, heat stress, and drought stress, as well as their combinations, providing an integrative overview of conserved and species‐specific metabolic stress responses in crops of agronomic relevance (Bulut *et al*., 2025b). These reports have identified the branched chain amino acids as one of the most responsive groups of metabolites. By integrating metabolomic data with physiological and omics analyses, researchers can gain a holistic understanding of plant adaptation mechanisms under future climate scenarios.

### 2. Hormonal regulation

**Fig. 1:**
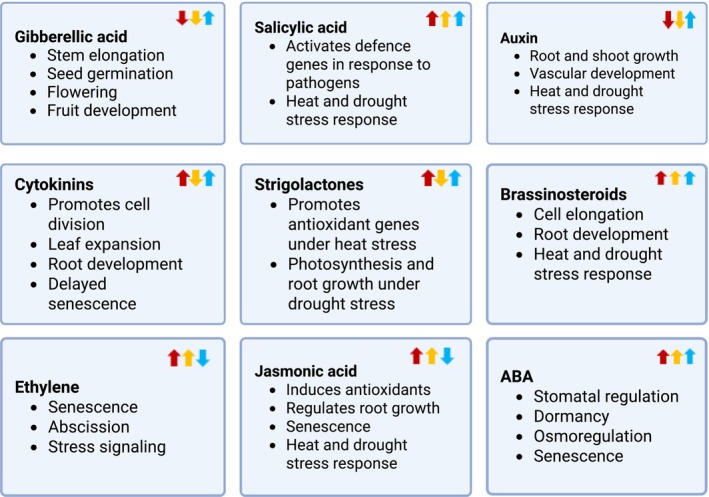
Phytohormone accumulation response and biological functions under heat, drought, elevated CO_2_. ABA, abscisic acid. The red arrow indicates heat stress, the orange arrow indicates drought stress, and the blue arrow indicates elevated CO_2_. By contrast, the direction of the arrow indicates an increase (up) or decrease (down) in the abundance of the phytohormone (Baxter *et al*., 2014; Ghosh *et al*., 2021; Jha *et al*., 2022; Roy & Mathur, 2021; Ullah *et al*., 2018; Table 2). This figure was created in BioRender. Pratap, A. (2026) https://BioRender.com/puw95k3.

Plant hormonal pathways coordinate growth, defence, and metabolic adjustments under environmental stress. Below, we summarise abiotic‐stress‐related responses of phytohormones. These hormones have several developmental roles, and these are important players in stress response or defence against pathogens (Fig. 1; Tables 1, 2).

ABA is a key mediator of plant responses to drought and other abiotic stresses, rapidly accumulating during water deficit to regulate stomatal conductance, osmotic balance, and protective gene networks (Table 2). ABA levels are strongly influenced by stress combinations: drought and heat together elevate ABA in wheat (Ahammed *et al*., 2021), whereas elevated CO_2_ often reduces ABA accumulation (Table 1). ABA also interacts with secondary metabolism, contributing to increases in phenolic acids, flavonoids, and sesquiterpenes under drought (Table 2). In thyme, moderate ABA restores terpene abundance that declines under drought (Table 2). Substantial genotypic and environmental variation in ABA dynamics suggests value as a metabolic biomarker for breeding climate resilience.

Auxin influences responses to heat, drought, and elevated CO_2_ by regulating developmental plasticity. Exogenous auxin improves reproductive outcomes during heat, increasing grain set in rice, and maintaining antioxidant capacity in pea (Table 2; Sergiev *et al*., 2018). Seed priming that enhances auxin biosynthesis improves drought tolerance via root development and seedling vigour (Table 2). Heat combined with elevated CO_2_ induces auxin‐mediated hyponasty in tomato (Table 2).

Jasmonic acid (JA) is a multifunctional regulator of abiotic tolerance. JA promotes thermotolerance by inducing antioxidants and heat shock proteins, stabilising membranes, and maintaining leaf water content (Rehman *et al*., 2023; Table 3). Drought strongly elevates JA, as shown by up to 10‐fold increases in tomato (Zhang & Huang, 2013). Elevated CO_2_ often suppresses JA biosynthesis and signalling (Table 2). Foundational pathway work established JA‐Ile as a major bioactive form (Clarke *et al*., 2009; Wasternack & Hause, 2013), and reviews outline the octadecanoid pathway (Feussner & Wasternack, 2002). JA responses are shaped by regulatory modules and crosstalk with other pathways (auxin/SnRK) that influence root development and drought tolerance (Table 2; Aggarwal *et al*., 2025).

**Table 3:** Comparison of different models for predicting agronomic traits using metabolome data.

Crops	Traits	Stress	Sources	BLUP	LASSO	OPLS	DNNGP	MLASSO	PLS	BayesB	RF	RKHS	SVM‐POLY
Rice	GY	D	G	0.35					0.35	0.35			
			G + M	0.59					0.34	0.59			
			M	0.56					0.54	0.55			
									0.64				
				0.38	0.46								
			G	0.38				0.39					
	Biomass			0.77				0.62					
	Leaf moisture			0.57				0.57					
	GY	C	G		0.16			0.19					
			T		0.49			0.25					
			M		0.46			0.23					
			M						0.417				
			G	0.16	0.19				0.12				0.17
			T	0.25	0.34				0.23				0.26
			M	0.2	0.17				0.21				0.23
			G + M	0.27	0.3				0.24				0.26
			G + T	0.22	0.19				0.23				0.24
			T + M	0.24	0.22				0.22				0.24
			G + T + M	0.26	0.23				0.23				0.24
	Amylose ratio		MB*			0.72							
	Ear emergence		MB*			0.65							
Maize	GY	D	G	0.31									
		H		0.33									
		D + H		0.21									
	KWPE	C	G	0.5			0.66						
			T	0			0.59						
	KN		G	0.47	0.36				0.44	0.46		0.47	0.48
			T	0.39	0.42				0.34	0.39		0.36	0.39
			M	0.38	0.41				0.32	0.29		0.41	0.3
	RN		G	0.58	0.53				0.57	0.6		0.58	0.58
			T	0.57	0.51				0.57	0.56		0.54	0.58
			M	0.51	0.46				0.47	0.31		0.51	0.35
Wheat	NGPS	C	M	0.48	0.51								
Potato	DRYM	D	T (*n* = 43)								0.7		
			T_s_ (*n* = 14)								0.67		
			M (*n* = 115)								0.92		
			M_s_ (*n* = 24)								0.9		
			T + M (*n* = 158)								0.83		
			T_s_ (*n* = 19) + M_s_ (*n* = 8)								0.78		
Sorghum	GY	D	G	0.36									
	SG			0.48									
Canola	GY	C	G	0.33									
			T	0.34									
			M	0.27									
			G + T	0.33									
			G + M	0.32									
			M + T	0.34									
			G + T + M	0.33									

Full references for this table are provided in Supporting Information Table S3. The selected parameters were chosen based on data availability and their relevance to breeding programmes. The blank cells represent data unavailability. BayesB, BayesianB; BLUP, best linear unbiased prediction; C, control conditions; D, drought; DNNGP, deep neural network‐based method for genomic prediction; DRYM, deviation of relative starch yield under control and drought conditions from its experimental median; G, Genomics; GY, grain yield; H, heat stress; HI, harvest index; MB*, multi‐block; KN, kernel number; KWPE, kernel weight per ear; LASSO, least absolute shrinkage and selection operator; M, metabolomics; MLASSO, multilayered least absolute shrinkage and selection operator; NGPS, number of grains per spike; OPLS, Orthogonal projections to latent structures; PLS, partial least square; RF, random forest; RKHS, reproducing kernel Hilbert space; RN, row number; SG, stay‐green; SVM‐POLY, support vector machine using the MLASSO polynomial kernel function; T, transcriptomics; _s_, selected/targeted. Scale**: 0 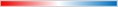 1.

* Multiblock/multiplatform data: capillary electrophoresis, gas chromatography, liquid chromatography, and ion trap, time of flight mass spectrometry.

** Scale values (0–1) denote prediction accuracy, with higher numbers indicating better performance (0.00–0.20: very low, 0.21–0.40: modest, 0.41–0.60: moderate, 0.61–0.80: strong, 0.81–1.00: very high). Because metrics, validation schemes, trait definitions, stress protocols, data preprocessing, and sample sizes differ across sources, values are best interpreted as within‐study comparisons. Any cross‐study comparisons should be made with caution because reported values may reflect different accuracy definitions (e.g. predictive *r* vs *R* ^2^) and cross‐validation designs.

Salicylic acid (SA) regulates defence‐related gene expression and contributes to abiotic stress responses. SA varies with genotype during drought and typically increases under heat but declines under elevated CO_2_ (Tables 1, 2). Combined stresses produce distinct patterns: SA often increases under heat plus drought but decreases under drought plus elevated CO_2_ (Tables 1, 2). Foliar SA application mitigates drought injury and improves yield in *Lallemantia Iberica* (Table 2).

CK levels decline during drought and heat due to reduced biosynthesis and enhanced degradation, leading to restricted growth and accelerated senescence (Table 2). Elevated CO_2_ stimulates CK biosynthesis and reduces degradation, particularly in leaves, enhancing photosynthesis and biomass (Table 2). Because CKs regulate meristems and roots, their modulation by contrasting stress factors has substantial consequences for resilience (Table 2).

Ethylene production rises rapidly with drought or heat, contributing to stress signalling, senescence, and resource remobilisation (Table 2; Fatma *et al*., 2022). Elevated CO_2_ commonly suppresses ethylene biosynthesis, delaying senescence, and altering stress sensitivity (Table 2; Pan *et al*., 2019). Ethylene also influences secondary metabolism under drought, for example via ethylene responsive factor‐mediated anthocyanin accumulation in *Melastoma spectabilis* (Xu *et al*., 2025; Tables 1, 2).

Gibberellic acid (GA) regulates elongation, reproduction, and germination but shows a stress‐dependent decline in endogenous levels. Exogenous GA offsets heat and drought effects, improving grain weight in wheat and enhancing drought tolerance in tomato (Nagar *et al*., 2021; Table 2). Endogenous GA often declines under heat, drought, and their combination, as in rice (Tables 1, 2). Elevated CO_2_ can compensate for reduced GA activity and rescue growth under GA inhibition (Table 2).

Brassinosteroids (BRs) support stress acclimation through growth control and transcriptional modulation. Under drought, BR‐responsive factors interact with drought regulators such as *RD26* to restrain growth when water is limiting (Table 2). BRs also remodel root architecture, including lateral root formation that can enhance water uptake (Gupta *et al*., 2020; Table 2). BRs further tune plant growth under stress by interacting with other phytohormone pathways (Table 2).

Strigolactones, belonging to terpenoid lactone hormones group, are known for their positively regulate abiotic stress tolerance, as shown in Arabidopsis (Tables 1, 2). Under drought stress, Strigolactones biosynthesis genes show increased expression, again pointing out their role in drought response, while their reduced synthesis leads to increased drought susceptibility due to limited root growth (Tables 1, 2; Zhong *et al*., 2024).

### 3. Crosstalk

**Fig. 2:**
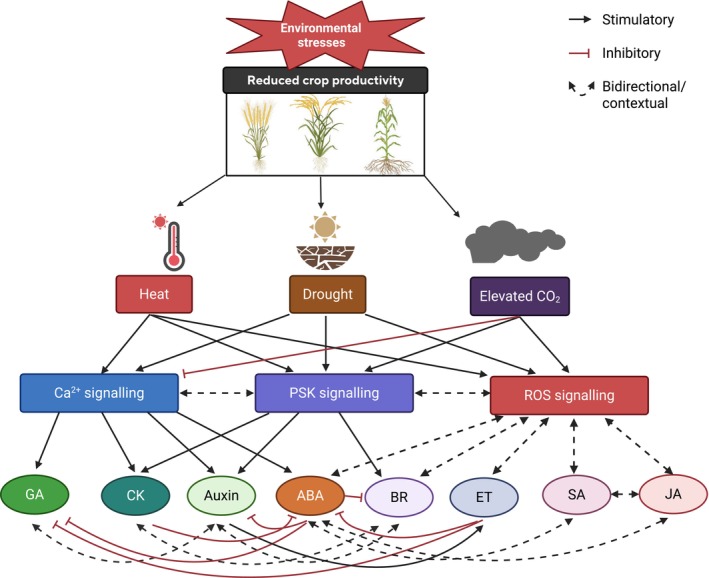
Crosstalk between phytohormones and signalling molecules under heat, drought, elevated CO_2_. ABA, abscisic acid; BR, brassinosteoids; Ca^2+^, calcium; CK, cytokinins; ET, ethylene; GA, gibberellic acid; JA, jasmoic acids; PSK, phytosulfokine‐ɑ; ROS, reactive oxygen species; SA, salicylic acid, Auxin (Baxter *et al*., 2014; Ullah *et al*., 2018; Ghosh *et al*., 2021; Roy & Mathur, 2021; Jha *et al*., 2022; Wu *et al*., 2025). This figure was created in BioRender. Pratap, A. (2026) https://BioRender.com/zgac9rr.

Plant signalling is a crucial component in initiating a response to abiotic stress. A vast network of proteins and metabolites is involved in orchestrating this crosstalk between environmental stressors, signalling molecules, and phytohormones. Calcium (Ca^2+^), phytosulfokine‐ɑ (PSK), and ROS are the signalling molecules that carry environmental cues from cell to cell through apoplastic and symplastic channels. This initiates downstream signalling pathways involved in plant growth and development through phytohormones. Combining reverse genetics approaches and metabolomics gives significant predictions on the crosstalk between phytohormones and other signalling molecules and their roles in stress response (Fig. 2; Table 2).

#### Phytohormones crosstalk

Several studies have highlighted the crosstalk between phytohormones, such as SA, ABA, JA, auxin, GA, ethylene, and BR under abiotic stresses (Jha *et al*., 2022; Table 2; Roy & Mathur, 2021). Crosstalk of BR with auxin and gibberellin further tunes growth under stress (Guo *et al*., 2024; Table 2). JA works synergistically with ethylene by inducing ethylene biosynthesis (Ghorbel *et al*., 2021), and under drought stress, an increase in the synthesis and metabolism of ABA and JA, as well as the activation of genes associated with the signalling pathways of both ABA and JA, is observed (Table 1). In rice and maize, it was shown that ABA regulates auxin biosynthesis at the root tip, thereby controlling gravitropic responses and root angle under drought conditions, which suggests that ABA could be utilised to enhance drought tolerance in cereal crops (Table 1). In wheat, the expression of the *TaFDL2*‐1A increased under drought stress and ABA treatment, which is associated with the *TabZIP8‐7A* protein, which enhances the plants' drought resistance. *TaFDL2*‐1A then binds to genes involved in auxin metabolism, such as *TaGH3.2*‐3A, *TaGH3.2*‐3B, *TaGH3.8*‐2A, and *TaGH3.2*‐2D within the ACGT core *cis*‐elements region, promoting their expression, which reduces auxin, ultimately impacting plant growth negatively (Table 2; Wang *et al*., 2019; Wang *et al*., 2024). Similarly, *PeFUS3* influences lateral root development by activating genes associated with auxin transport, such as PIN‐FORMED (*PIN*)*2*, *PIN6a*, and AUXIN RESISTANT 1 (*AUX1*). It also upregulates *PePYL3*, which enhances drought tolerance. *PeFUS3* further interacts with the ABA‐responsive TF *PeABF2*, linking it to the ABA signalling pathway (Li *et al*., 2025). In carrots, the storage roots are crucial for early drought stress detection, triggering the expression of specific genes, such as *DcNCED3*, which is involved in ABA biosynthesis, and *DcYUCCA6*, which is linked to auxin production. These hormones help the plant increase the production of metabolites, such as proline, GABA, and sugars, which are vital for effectively managing drought stress (Table 1). Auxin is also essential for the formation of leaf venation through the action of the *PIN1* efflux carrier, which plays a critical role in vascular function and drought resistance (de Carvalho *et al*., 2025). To adapt to drought stress, plants enhance sugar accumulation and water transport systems. In bamboo, the genes *PeSTP*_46019 and *PeSWEET*_23178 are activated by the TF *PeHDZ72*, which increases drought tolerance by enhancing sugar transport. Additionally, the *PeTIP4*‐3 gene contributes to drought resilience by improving water transport (Zhu *et al*., 2025). Phytohormones are highly responsive to environmental changes and significantly contribute to plant growth, development, and productivity, making them effective metabolic biomarkers for climate resilience in crops.

The sessile nature of plants requires environmental sensing through signalling molecules and downstream pathways to defend themselves against environmental stressors. Signalling molecules play an important role in various cellular processes, and hence, their role in metabolic regulation and crosstalk is pivotal (Fig. 2). Under heat stress, there is a change in the cytosolic concentration of early signalling molecules such as Ca^2+^ and ROS (Adem *et al*., 2020; Kan *et al*., 2022).

##### ROS signalling

In response to environmental stressors, plants generate a variety of metabolites during the early stages of stress through a complex regulatory system that links external stimuli to molecular signalling pathways. This system includes the rapid production of ROS and the formation of ROS waves that propagate long‐distance signals through apoplastic and symplastic channels, enabling systemic coordination of stress responses. These ROS‐mediated waves integrate local and distal signalling, modulate developmental processes, and interact with hormonal and calcium signalling networks to shape whole‐plant acclimation under fluctuating environmental conditions (Baxter *et al*., 2014; Schieber & Chandel, 2014; Mittler *et al*., 2022; Waadt *et al*., 2022). ROS act as a vital signalling molecule that regulate numerous biological processes in plants, such as growth, development, and responses to both biotic and abiotic stress (Baxter *et al*., 2014). In plants, antioxidant enzymes are essential for maintaining levels of ROS, including their production, scavenging, and transport. Respiratory burst oxidase homologues (*RBOHs*) are key signalling nodes in these regulatory networks (Suzuki *et al*., 2011). For instance, the *OsRbohB* gene in rice is responsible for ROS production, playing a critical role in helping rice plants manage drought, heat, and salinity by influencing growth and hormonal balance (Chen *et al*., 2024; Shi *et al*., 2020). BRs, ROS, and phenylpropanoid pathways interact closely to coordinate plant defence and stress tolerance under abiotic stress conditions (Table 2). Proline accumulation is also linked to various stress responses, developmental processes, and the size of root meristems. The interplay between proline and ROS emphasises a complex regulatory framework that is crucial for plant resilience and survival (Renzetti *et al*., 2025). Furthermore, the regulation of ROS production by the rice kinase *OsMRLK63* is essential for enhancing drought tolerance (Table 2). In response to UV stress, *Pinus radiata* initially mitigate UV toxicity by reducing photosystem activity and the activity of the electron transport chain. These responses involved the accumulation of protective components and an increase in photorespiration, which helps lower ROS levels. New protease and kinase proteins have been identified as playing roles in signalling pathways and the regulation of the UV stress response (Pascual *et al*., 2017). ROS accumulation and ROS signalling waves generated under environmental fluctuations form a pivotal regulatory layer in plant stress management. They shape developmental outcomes, modulate growth, and enhance the plant's ability to withstand diverse environmental challenges, thereby contributing significantly to climate resilience.

##### Calcium signalling

Calcium signalling is one of the earliest responses to environmental stimuli. ROS and Ca^2+^ often work together in the propagation of environmental cues and control plant growth, development, and defence. Environmental stresses are relayed through an inward Ca^2+^ flux, initiating a stress response through *RBOH* that generates a ROS burst (Ravi *et al*., 2023). The opening of Ca^2+^ channels through a ROS receptor *HPCA1* leads to stomatal closure (Wu *et al*., 2020). In Arabidopsis, Ca^2+^ influx due to heat stress activates CALMODULIN (*CAM*)*1* and *CAM3*, which in turn increase the expression of HEAT SHOCK TRANSCIRPTION FACTOR (*HSFA*)*2*. The *HSFA2* regulates the expression of LIPOXYGEANSE (*LOX3*) and 12‐OXOPHYTODIENOATE REDUCTASE 3 (*OPR3*) genes, facilitating the accumulation of JA (Guo *et al*., 2024). Together with ROS signalling, Ca^2+^ signalling enables plants to initiate a response to multifactorial abiotic stresses through a series of downstream pathways and modulate phytohormone levels (Haider *et al*., 2022).

##### PSK signalling

PSK is a pentapeptide known for its role in promoting cell elongation and primary root growth, which plays a significant role in mitigating drought stress. In Arabidopsis, PSK receptors and PSK genes modulate root growth and provide drought tolerance (Stührwohldt *et al*., 2021). In tomato, PSK signalling controls drought‐induced flower abscission (Reichardt *et al*., 2020). PSK receptors are known to activate diverse signalling cascades, such as cGMP‐dependent signalling, phosphorylation events, Ca^2+^ signalling, MITOGEN ACTIVATED PROTEIN KINASE (*MAPK*) pathways, and transcriptional regulation (Wu *et al*., 2025).

### 4. Temporal dynamics of metabolite responses

Drought and heat stress trigger a highly dynamic and time‐sensitive reprogramming of metabolism, as plants strive to maintain cellular homeostasis and survive under adverse conditions. In the early stages of stress exposure, a rapid accumulation of osmoprotective metabolites, such as proline, soluble sugars, and specific amino acids, occurs, which helps stabilise proteins and cellular structures while maintaining osmotic balance (Li *et al*., 2024). As stress persists, the metabolic profile shifts, with a gradual increase in secondary metabolites, including flavonoids, phenolic acids, and antioxidants (Sun & Fernie, 2024). These compounds play crucial roles in scavenging ROS and protecting cells from oxidative damage (Salam *et al*., 2023; Sun & Fernie, 2024). Over time, the coordinated buildup of both primary and secondary metabolites reflects the plant's transition from immediate protective responses to the establishment of long‐term defence strategies (Salam *et al*., 2023). This temporal orchestration of metabolic changes enables plants to optimise their physiological responses, balancing rapid adaptation with sustained resilience during prolonged periods of drought and heat stress (Zinta *et al*., 2018).

Elevated CO_2_ stress triggers a sequence of time‐dependent metabolic adjustments in plants, reflecting a sophisticated strategy to balance immediate growth with long‐term resilience. In the initial phase of exposure, increased photosynthetic carbon assimilation leads to a rapid accumulation of primary metabolites, including soluble sugars and amino acids (Zinta *et al*., 2018; Zhao *et al*., 2020). This metabolic surge supports accelerated growth, energy storage, and cellular maintenance. As the exposure continues, the plant's metabolic profile gradually shifts toward the production of secondary metabolites. These compounds accumulate more slowly but are critical for protecting cells from oxidative damage and modulating stress signaling pathways. Over days to weeks, the continued rise in these protective metabolites marks a transition from growth promotion to the establishment of robust defence mechanisms. This dynamic and temporal reprogramming of plant metabolism under elevated CO_2_ underscores their capacity to adapt both immediately and sustainably to a changing environment (Zhao *et al*., 2020).

## IV. Quantitative biomarker panels for stress prediction

### 1. Biomarker discovery and validation

NMR and MS are two widely used metabolomics techniques in plant sciences. While both have their benefits and limitations, NMR presents structural information and absolute quantification, whereas MS offers higher analytical sensitivity, providing a more detailed survey of the entire metabolome. Both NMR and MS can be employed in a targeted or nontargeted approach, depending on the research question; however, MS requires labelled isotopes for absolute quantification. MS is often coupled with a variety of separation techniques, including gas chromatography (GC) and liquid chromatography (LC), and offers higher analytical sensitivity, enabling the detection and quantification of thousands of metabolite peaks. It is especially well‐suited for complex biological samples (Rhee & Gerszten, 2012). However, one significant advantage of NMR is that it does not require sample destruction. For example, a custom‐made ^13^C/^1^H double‐resonant coil enabled the noninvasive imaging and monitoring of sucrose allocation within the seed, providing movement of ^13^C‐sucrose at a submillimetre level of resolution (Rapley & Whitehouse, 2015; Paniagua‐Michel & Olmos‐Soto, 2016; Aina *et al*., 2024). Although NMR provides a real‐time, nondestructive assay for metabolites, often the benefits of using this technique are overshadowed by ambiguity. A triple quad MS offers high sensitivity and the ability to confirm specific precursor/product ion combinations for known metabolites. This approach with a targeted list of metabolites offers much higher confidence in the identity of metabolites using collision‐induced‐dissociation analyses of commercial standards (Rhee & Gerszten, 2012). Hence, a selection of metabolites for targeted approaches provides greater sensitivity and confidence.

Plants have developed complex mechanisms for rapidly detecting and efficiently adapting to environmental stressors. Biomolecular condensates play a crucial role in regulating stress perception and response in plants. These condensates employ various mechanisms to regulate essential cellular processes, including transcription, translation, RNA metabolism, and signalling pathways, particularly under stress conditions (Peng *et al*., 2025).

The emergence of MS has enabled the development of biomarkers associated with both environmental and biological stresses. These biomarkers are quantifiable and consistent, providing valuable insights into how plants respond to various biotic and abiotic factors. They provide a fast and efficient way to identify and manage stressors in plant systems (Rapley & Whitehouse, 2015; Paniagua‐Michel & Olmos‐Soto, 2016; Aina *et al*., 2024). For example, using *metQTLs*, a study on 385 maize lines under normal and drought conditions identified 3890 metabolites, with 1035 linked to drought responses and tolerance. Additionally, *Bx12* and *ZmGLK44* were identified as genetic markers associated with drought, playing a role in regulating metabolite production and improving drought resilience (Zhang *et al*., 2021). The metabolome analysis identified four key biomarkers in response to drought, enhanced nitrogen recycling through purine and pyrimidine metabolism, drought‐induced senescence marked by the accumulation of 1‐aminocyclopropane‐1‐carboxylic acid and asparagine, and a protective antisenescence response driven by serotonin accumulation during severe drought stress (Table 1). Indeed, drought stress during seed development results in seed shrinkage, with less accumulation of essential substances such as sugars and proteins in sensitive wheat lines compared to drought‐tolerant varieties. Genes related to protein and starch production are also less active in drought‐sensitive wheat, suggesting that improved drought‐resistant traits could help enhance seed formation under dry conditions (Table 2). Aminotransferases play a critical role in helping plants survive challenging conditions, with *GhTAT2* genes particularly enriched in pathways related to the metabolism of tyrosine, cysteine, methionine, and phenylalanine, as well as the biosynthesis of various alkaloids, including isoquinoline, tyrosine, tryptophan, tropane, piperidine, and pyridine (Table 1).

Metabolomics provides a powerful tool for identifying biomarkers that predict crop stress tolerance under elevated CO_2_ conditions. Increased CO_2_ alters the abundance of key metabolites, including organic acids, sugars, and amino acids, which are crucial for stress resilience and maintaining energy balance. For example, malate and fumarate accumulation under elevated CO_2_ have been linked to enhanced carbon assimilation in wheat, whereas proline accumulation indicates an osmoprotective response in soybean (Malhi *et al*., 2021). Furthermore, metabolic reprogramming under elevated CO_2_ modulates antioxidant pathways and secondary metabolite production, influencing plant defence mechanisms (Singh & Agrawal, 2015). By incorporating these metabolic signatures into biomarker panels, researchers can enhance predictions of crop performance, nutrient use efficiency, and stress resilience, underscoring the importance of metabolomics‐driven screening in breeding programmes. Elevated CO_2_ also induces accumulation of phosphorylated sugar intermediates, starch and flavonoids (Levine *et al*., 2008) and in rice, under combined heat and drought stress, yield stability positively correlated with arabitol, glucose‐6‐phosphate, and sucrose at the flowering stage, and at early grain filling with succinic and threonic acids while it negatively correlated with dehydroascorbic acid (Table 1). Studies of the response of maize under combined heat and drought conditions show a decrease in shikimate, glucose, and sucrose while an increase in phenylalanine, fumarate, choline, asparagine, aspartate, malonate, citrate, GABA, isobutyrate, valine, and isoleucine (Table 2). There is considerable variability in environmental‐induced changes in metabolite abundance, depending on genotype, mode, and intensity of stress exposure, as well as developmental stage and tissue type (Table 1). This variability makes it challenging to adopt these metabolites as biomarkers universally and needs to be considered when designing an experiment to ensure the desired level of transferability can be achieved. These studies clearly show that metabolite responses can be utilised to assess plant response to abiotic stresses, and there is a significant opportunity for using them as a selection tool in breeding. Metabolic profiling of young plants grown under controlled environment conditions offers predictive ability for important agronomic traits. For example, selection through untargeted screening revealed that carbohydrates, sugar alcohols, and GABA in young chickpea plants under well‐watered conditions could predict seed number (*R* ^2^ = 0.62) under terminal drought (Purdy *et al*., 2023). In wheat, under heat stress, DM2 domain‐containing protein (*r* = 0.99, *P ≤* 0.05) and Rubisco activase (*r* = 0.96, *P ≤* 0.05) correlated between seedling and ear peep stages, opening a new portal for early detection of flowering stage heat tolerance (Pratap *et al*., 2026). Untargeted metabolomics also presents an excellent opportunity for the discovery of metabolic biomarkers. For example, the metabolic profiling of tomato under heat stress identified 22 discriminatory metabolite biomarkers associated with enhanced antioxidant activity (Singh *et al*., 2024). Similarly, metabolic biomarkers have been identified for yield stability and seed quality under combined heat and drought stress in rice (Table 1). In wheat, genetic drought tolerance was strongly predicted by glasshouse relative water content (RWC), which correlated with yield gap‐based drought tolerance (*r* ^2^ = 0.85, *P* < 8 × 10^−6^) and field RWC (*r* ^2^ = 0.77, *P* < 0.05), while drought‐induced changes in four metabolites, serine, asparagine, methionine, and lysine, explained 98% of genetic yield gap‐based drought tolerance variation (*R* ^2^ = 0.98, *P* < 0.01) (Table 1).

### 2. Predictive power over mechanistic understanding

Metabolomics generates vast amounts of data, providing essential mechanistic insights into plant responses to abiotic stressors. This diversity of data can be harnessed alternatively for the prediction of plant phenotype. The addition of metabolomics data to already existing GP models is already presented in the current literature. Researchers have also explored the independent ability of metabolomics data in assessing the yield stability of crops. For example, in cucumber, elevated phytohormones such as zeatin riboside, BRs, and JA also correlated positively with thermotolerance (Table 1). However, selection of metabolites based on known biological pathways for that specific condition may not increase the predictive value. This point has been proven empirically in clinical studies (Pepe & Thompson, 2000).

Metabolomics panels are rapidly emerging as a cornerstone for predicting plant stress responses, offering a level of predictive power that often surpasses what can be achieved through traditional mechanistic understanding alone. By capturing the comprehensive metabolic fingerprints of plants under drought, heat, elevated CO_2_ and other abiotic stresses, these panels provide a holistic snapshot of the physiological state that integrates signals from multiple pathways and regulatory networks. This approach enables researchers to identify robust biomarkers that serve as early warning indicators, allowing for timely intervention and more precise selection in breeding programmes (Karakas *et al*., 2025). The predictive strength of metabolomics panels is further enhanced when integrated with other omics layers, as demonstrated in the multi‐omics study by Guo *et al*. (2016), in which combining metabolite profiles with genomic and transcriptomic data significantly improved the accuracy of yield predictions under drought conditions in maize. These panels are constructed using high‐throughput analytical techniques and advanced computational models, such as ML, which can sift through vast datasets to pinpoint the most informative metabolites for stress prediction. Significantly, the success of these panels does not always depend on a detailed mechanistic understanding of each metabolite's functional role; instead, their value lies in their empirical association with stress outcomes across diverse genotypes and environments. Metabolomics‐driven prediction can outperform pathway‐based approaches, especially when the underlying biological mechanisms are complex or not fully elucidated.

### 3. Integration with AI and high‐throughput screening

**Fig. 3:**
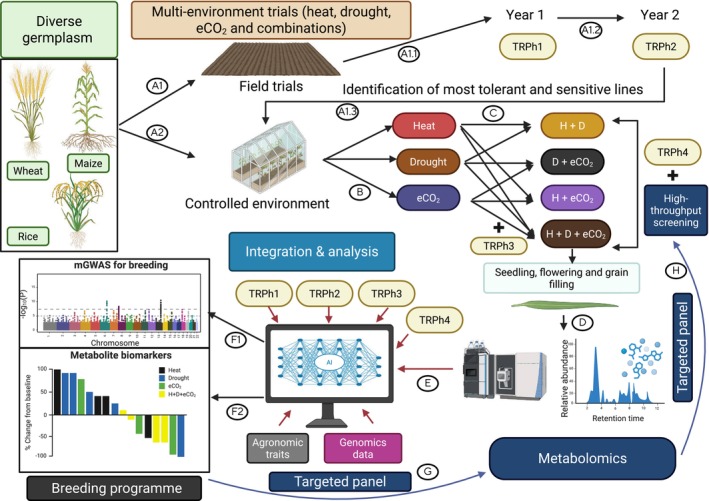
Summarised methodology for metabolite biomarker discovery and genomic targets selection for those metabolites to predict high‐throughput phenotypic and agronomic traits of interest for direct uptake in breeding programmes. D, drought; eCO_2_, elevated CO_2_; H, heat; mGWAS, metabolome‐wide genomic association studies; TRPh 1–4, time‐resolved phenotyping with numbering for each set of phenotyping data. Steps A–B represent the flow in this figure. This figure was created in BioRender. Pratap, A. (2026) https://BioRender.com/8dpw5w7.

Including the predictive ability of metabolomics in current plant breeding programmes remains a challenge in developing climate‐resilient crops. In potatoes, high‐throughput phenotyping combined with multi‐omics provided an in‐depth understanding of multi‐abiotic stress resilience, including heat, drought, heat‐drought, and waterlogging (Table 1). Additionally, metabolomics and high‐throughput phenotyping have provided deeper insights into the melatonin‐assisted alleviation of salt stress in lettuce and the screening of biostimulants to increase tomato productivity (Paul *et al*., 2019; Secomandi *et al*., 2025). AI facilitates the integration of multiple large datasets, providing a more comprehensive picture of the stress response in plants (Table 1). Hence, a combination of metabolite biomarker discovery and genomic targets for those metabolites to predict high‐throughput phenotypic and agronomic traits of interest can be directly integrated in breeding programmes for climate‐resilient crops. However, a stepwise process needs to be followed in order to realise the accurate potential of these techniques, which includes field trails to find cultivars with contrasting tolerance multiple stresses. The controlled environment testing of these cultivars would provide a robust set of biomarkers, which can be reconfirmed with a targeted panel. The practice of trying different developmental stages and tissue types helps in pinpointing the exact scenario for better prediction of complex traits while maintaining high throughput (Fig. 3).

## V. Advanced prediction models for stress tolerance

### 1. Genomic and metabolic marker‐assisted breeding

GP is a statistical approach that predicts phenotypes by utilising genome‐wide markers, potentially accelerating molecular plant breeding. However, the progress relying solely on genomic data for phenotype prediction has plateaued. Recent research has highlighted transcriptomic prediction (TP) and metabolomic prediction (MP) as promising alternatives for predicting gene expression and metabolic pathways. Despite their potential, both TP and MP currently overlook genomic data, which plays a crucial role in phenotype determination, especially for traits with high heritability (Table 3; Meuwissen *et al*., 2001). There are three primary types of statistical methods used in GP: parametric methods such as Best Linear Unbiased Prediction (GBLUP) (Xu *et al*., 2014) and Least Absolute Shrinkage and Selection Operator (LASSO) (Li & Sillanpää, 2012), semiparametric methods such as Reproducing Kernel Hilbert Space (RKHS) (Gianola *et al*., 2006), and statistical learning methods, including Support Vector Machines (SVM) (Maenhout *et al*., 2007), Neural Network (Gianola *et al*., 2011), and Random Forest (Holliday *et al*., 2012). Recently, the Multilayered Least Absolute Shrinkage and Selection Operator (MLLASSO) has emerged as an advanced method that integrates data from metabolomics and transcriptomics layers into a single model (Table 3). MLLASSO improves the predictability of rice yield by capturing higher order gene interactions, thereby boosting prediction accuracy from 0.1588 (using GP alone) to 0.2451 with MLLASSO (Table 3). The RR‐BLUP model was used to estimate the performance of maize genotypes for various traits under both optimum and drought‐stress conditions. The prediction accuracies were moderate to high for all eight traits under optimum conditions, and low to moderate under drought conditions (Amadu *et al*., 2025).

Metabolomics links genotype and phenotype, enabling researchers to accurately predict plant performance under stress conditions. The metabolic signatures obtained through metabolomic approaches can serve as biomarkers, providing early indicators of stress tolerance and agronomic performance (Table 1). Combined with GP models, metabolomics can significantly enhance the predictive ability of these models. Partial least squares regression (PLSR) is one of the most frequently used models in metabolomics to correlate metabolic profiles with specific phenotypic traits. Previously, PLSR models have been extensively used to link metabolites to agronomic traits in rice and maize (Dan *et al*., 2021; Table 3). Several studies have included BLUP models combining GS with metabolomics data for predicting conventional traits in rice, maize, oats, and foxtail millets (Hu *et al*., 2021; Schrag *et al*., 2018; Table 2; Wei *et al*., 2023). In oats, metabolomics BLUP outperformed transcriptomics BLUP and genomics BLUP when combined with SNPs in predicting 17 traits in two oat breeding populations, especially in multi‐environment and multi‐trait datasets (Hu *et al*., 2021). BLUP has outperformed the LASSO method on multiple occasions in predicting traits that combine genomics and metabolomics data (Table 3; Yang *et al*., 2025). However, LASSO offers superior prediction power and lower false positives compared with linear regression, linear mixed models, and Bayesian analysis (Wei *et al*., 2018).

### 2. Machine learning models

AI, particularly ML, significantly enhances the predictive capabilities of metabolomics by handling complex, multidimensional datasets. ML models, such as random forests, neural networks, and SVMs, are increasingly applied to predict crop performance under stress. For example, Random Forest models, integrating multiple decision trees, efficiently identify essential metabolites as predictors of plant traits. Random Forest algorithms have been applied to predict yield stability in rice under field‐based combined drought and heat stress, identifying metabolic predictors with high accuracy (Table 1). Additionally, deep learning approaches, such as artificial neural networks, provide superior predictive accuracy by modeling nonlinear relationships within metabolomics data. Recent studies suggest that deep learning could further enhance the prediction of complex traits, such as stress tolerance and yield potential, from metabolite profiles (Table 1). Moreover, SVMs have been used for classification tasks, distinguishing between stress‐tolerant and ‐sensitive cultivars based on metabolomic signatures. In cucumber, SVM successfully predicted thermotolerance levels from metabolic biomarkers, demonstrating the utility of metabolomics‐AI integration (Table 1).

### 3. Model validation metrics

In predictive modelling for plant breeding using genomic, transcriptomic, and metabolomic data, model validation is essential to assess the reliability and generalisability of predictions. A variety of validation metrics and strategies are used to evaluate the predictive performance of models across different environments, traits, and stress conditions (Bhatta *et al*., 2020; Table 3). Cross‐validation is the most widely used strategy for estimating prediction accuracy in genomic and metabolomic prediction models (Hoque *et al*., 2024). There are various metrics, often used in conjunction, to demonstrate the effectiveness of a model. For example, Pearson's correlation coefficient (*r*) between predicted and observed phenotypic values is a primary metric, coefficient of determination (*R* ^2^) measures the proportion of variance in phenotype explained by the model, and mean squared error (MSE) and root MSE (RMSE) measure the average deviation between predicted and actual values (Bhatta *et al*., 2020; Haile *et al*., 2021; Table 3). Whenever possible, independent test sets (from different years, environments, or breeding programmes) are used to test the model's robustness and avoid overfitting, which is especially critical in metabolomics in which high‐dimensionality can lead to model overfitting (Table 3; Wang *et al*., 2019; Yang *et al*., 2025).

## VI. Conclusion and future perspectives

### 1. Metabolomic panels for abiotic stress tolerance

The integration of metabolomics data with AI and ML platforms promises advanced predictive precision for plant breeding. Future research should leverage an integrative ‘omics’ approach, combining metabolomics and genomic selection, to build comprehensive predictive models. Developing user‐friendly AI‐driven analytical software platforms will also democratise predictive metabolomics in practical agricultural settings. Multiple studies have investigated transcriptomics, metabolomics, and genomics individually and in combination to assess the predictive capabilities of various models for agronomic traits, and it is quite established that in some cases, especially when predicting yield stability, the integrative multi‐omics have the upper hand over conventional GP models (Table 3). Across crops and traits, the table shows that prediction accuracy varies widely depending on the stress condition, data source, and model used. In many cases, combining genomics and metabolomics improves accuracy compared with using either alone, especially for rice grain yield under drought or several maize kernel traits. Nonlinear models such as DNNGP, RF, RKHS, and SVM often outperform simpler linear methods for more complex traits. Metabolomics can be particularly powerful for stress‐related traits, as seen in potato drought performance, in which metabolite‐based models achieved the highest accuracy. However, improvements are not universal, as some crops such as canola show similar prediction accuracy across all omics combinations. Overall, the table highlights that model performance is highly trait‐ and crop‐specific, and integrative multi‐omics approaches often, but not always, provide the greatest benefit. However, there is a lack of standardised study design to identify and validate the omics results in a broader context of stress response.

### 2. Bridging molecular insights with practical breeding and crop management

Metabolomic analysis provides crucial targets for functional characterisation and often leads to a deeper understanding of biological processes. These targets are crucial to the development of stress‐resilient crop varieties. Using these targets for predicting important agronomic traits provides a pathway for more accurate prediction of complex traits that are not directly explained by genomic data. Metabolomics provides a link between the genotype and the phenotype under environmental variability, significantly improving earlier predictive models that relied on genomic data only. Current breeding practices heavily rely on genomic selection of varieties, but genomics has its limitations. Hence, creating new workflows by integrating metabolomics in those breeding programmes will be a significant leap forward towards a more sustainable increase in crop productivity under climate change.

### 3. Future directions and research integration

Metabolomics, supported by advanced analytical tools, statistical modelling, and AI methods, offers powerful capabilities for predicting plant traits under heat, drought, elevated CO_2_, and combined heat‐drought‐elevated CO_2_ stresses. The synergistic integration of metabolomics with ML and deep learning can significantly accelerate breeding efforts and the development of stress‐resilient crop varieties. Continued advancements in analytical methodologies and computational tools will strengthen the role of metabolomics as an indispensable resource for agriculture in the face of climate uncertainties.

### 4. Enhancing global food security through omics innovations

Currently, research in the field of agriculture is evolving rapidly in the era of data‐driven decision‐making and research and development. Advancements in remote sensing, imaging, monitoring, biotechnology, and molecular biology all significantly contribute to the agriculture industry. The Omics technologies provide enormous data that hold the secrets of system biology, yet it is highly resource‐intensive and expensive. The cost of running multi‐omics analysis is decreasing due to significant technological advancements. This has provided an opportunity for integrating this technology into current plant breeding programmes around the world. Major growth in crop productivity will come from genetic improvements, and omics will play an essential role in linking crop phenotypes with genotypes under a changing climate, thereby ensuring global food security.

### 5. Importance of cross‐disciplinary collaborations

Although omics technologies offer deeper insights into genotype × environment interactions, these techniques can yield different results under varying management regimes due to their high sensitivity to environmental cues. There is a need for more accurate data on agronomic traits, such as developmental stage, weather conditions, soil nutrient availability, and microbiome. This breakdown of genotype × environment × management interactions requires more collaborative research project designs involving multiple aspects of agricultural research, such as agronomy, physiology, plant pathology, genetics, and plant breeding, as well as high‐throughput phenotyping, engineering, and data science. With careful consideration of all the above aspects, the true potential of these highly sensitive and specific technologies can be realised for breeding climate‐resilient crop varieties. The selection of germplasm panels for this analysis is equally important, as it determines the mapping power for identifying genetic markers. Metabolite abundance changes under variable environments can also serve as a proxy for genetic diversity and act directly as biomarkers for complex traits, such as climate resilience. However, the predictive ability depends on the diversity of germplasm selected during the development of the panels. Hence, global panels offer more transferability of findings but require collaborations across international institutions.

## Competing interests

None declared.

## Disclaimer

The New Phytologist Foundation remains neutral with regard to jurisdictional claims in maps and in any institutional affiliations.

## Supporting information


**Table S1** All references for metabolite abundance changes compared to ambient conditions under heat, drought and elevated CO_2_ and their combinations (Table 1).
**Table S2** All references for overview of phytohormone biosynthesis, signalling, and functional roles under environmental stresses (Table 2).
**Table S3** All references for comparison of different models for predicting agronomic traits using metabolome data. The selected parameters were chosen based on data availability and their relevance to breeding programmes (Table 3).
Please note: Wiley is not responsible for the content or functionality of any Supporting Information supplied by the authors. Any queries (other than missing material) should be directed to the *New Phytologist* Central Office.
